# Comment on ‘Duloxetine for rehabilitation after total knee arthroplasty: a systematic review and meta-analysis’

**DOI:** 10.1097/JS9.0000000000000567

**Published:** 2023-06-22

**Authors:** Meiru Chen, Dandan Zhang, Wenhui Hu, Xiumei Tang, Xiaoling Hou

**Affiliations:** a Department of Orthopaedics, West China Hospital, Sichuan University/West China School of Nursing; b Trauma Center, West China Hospital; c Department of Respiratory and Critical Care Medicine, Med-X Center for Manufacturing, Frontiers Science Center for Disease-Related Molecular Network, West China Hospital, West China School of Medicine; d Health Management Center, General Practice Medical Center, West China Hospital, Sichuan University/Institute of Hospital Management, West China Hospital, Sichuan University, Chengdu, People’s Republic of China

*Dear Editor,*

Yang *et al*.^1^ recently performed an interesting systematic review that evaluated the efficacy and safety of duloxetine for postoperative recovery after total knee arthroplasty (TKA). Their findings revealed that duloxetine showed a statistically significant reduction in pain at rest (at 3 days, 1 week, 2, and 6 weeks), pain on movement (at 5 days, 1 week, 2, 4, 6, and 8 weeks), and significant improvement in physical function, range of motion of the knee at 6 weeks, emotional function (depression and mental health), and cumulative opioid consumption at 24 h. The findings provide important evidence for the treatment strategy of TKA patients. However, some concerns still need further clarification.

First, the results section is methodologically incorrect. The author reported that they included 11 randomized controlled trials (RCTs) studies. However, 4 out of the 11 studies are inappropriate. (i) The study of Kim *et al*.^2^ is a retrospective study, as described in the methods part, which should not be included. Thus, the quality of Kim *et al*.’s study should be re-evaluated using the Newcastle–Ottawa Scale (NOS) for the retrospective study. The author could either perform a subgroup analysis or remove the study from the pooled results since different research designs could increase heterogeneity. And also, this meta-analysis did not investigate the sources of study heterogeneity. (ii) In the study of Liu *et al*.^3^, they recruited 66 patients, and all of them had depression when they underwent TKA, which contract this review’s exclusion criteria as ‘*patients who had known abnormal liver and renal function, an allergy to duloxetine, or a psychiatric disorder*’. And we strongly suggest this article be removed since it would insert bias into the overall results because of the antidepressant nature of duloxetine. Additionally, the outcomes of Liu *et al*.^3^ reported were HAMD-17, WOMAC, and KSS, whereas the author mistakenly used WOMAC scores as VAS scores in the pooled results. Regrettably, this processing is highly inappropriate and should be corrected. (iii) In another study by Wang *et al*.^4^, they used Qing Peng ointment (a traditional Chinese medicine) and duloxetine as a drug combination, which apparently did not meet the inclusion criteria ‘*RCTs comparing duloxetine with placebo, no duloxetine, or no duloxetine intervention*’. Moreover, the outcomes Wang *et al*.^4^ reported were visual analogue scale (VAS), WOMAC, IL-1, IL-6, and TNF-α, which were not consistent as the author reported in this meta-analysis. The above information indicates that there exist unignorable flaws in the process of article selection, data extraction, and data analysis, which affect the ultimate findings of this review. We would highly recommend that the author recheck the accuracy of the original data.

Second, the author only listed the type of complications but reached the conclusion that there were no significant differences in the incidence of adverse events between the duloxetine groups and controls. However, after meta-analysis, we found there were substantial differences. Six studies involving 522 patients (260 in the duloxetine group, 262 in the placebo group) showed that duloxetine reduced the rate of nausea/vomiting (Log RR, −0.48; 95% CI, −0.95 to −0.00; *I* ^2^=10.73%) but increased the rate of drowsiness (Log RR, 0.66; 95% CI, 0.22–1.29; *I* ^2^=16.37%) (Fig. 1).

**Figure 1:**
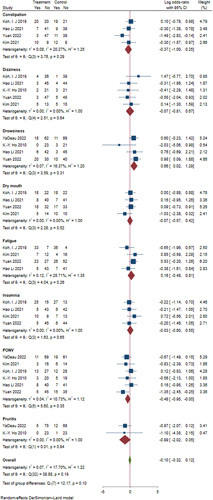
The forest plot of complications (duloxetine versus control)

Third, there are studies that investigate the effect of duloxetine in patients with central sensitization (CS). The baseline characteristics were unbalanced, leading to incomparability between groups. Considering CS is an important consideration before routinely using duloxetine, the author neither presented the baseline information of included patients nor discussed the effects, which we would like to supplement in this letter. We found duloxetine administration was associated with statistically significant lower ambulation pain scores in both CS patients (reported in three studies: MD, −0.59; 95% CI, −0.87 to −0.31; *I* ^2^=82.78%) and patients with n-CS (reported in five studies MD, −0.36; 95% CI, −0.46 to −0.27; *I* ^2^=42.02%) (Fig. 2).

**Figure 2:**
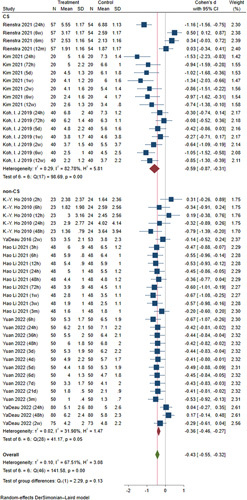
The forest plot of ambulation pain scores in CS and non-CS subgroups (duloxetine versus control)

We sincerely appreciate the authors’ contribution to the study, as the field of multimodal pain management is rapidly evolving. We recommend that the authors take into consideration the mentioned issues to ensure the quality and reliability of this research endeavour.

## Ethical approval

Not applicable.

## Consent

Not applicable.

## Sources of funding

None.

## Author contribution

C.M.R. and Z.D.D.: study design and writing; H.W.H., T.X.M., and H.X.L.: language polishing and final approval.

## Conflicts of interest disclosure

All authors declared that there are no conflicts of interest.

## Research registration unique identifying number (UIN)

Not applicable.

## Guarantor

Tang Xiumei and Hou Xiaoling.

## Data availability statement

The data that support the findings of this study are available from the corresponding author upon reasonable request.

## Provenance and peer review

Commentary, internally reviewed.
